# The detection of cannabinoids in breath after ingestion of cannabis-infused edibles

**DOI:** 10.1093/jat/bkaf063

**Published:** 2025-07-10

**Authors:** Jennifer L Bery, Ashley Brooks-Russell, Tara M Lovestead, Kavita M Jeerage

**Affiliations:** Applied Chemicals and Materials Division, National Institute of Standards and Technology, 325 Broadway Ave, Boulder, CO 80305, United States; Colorado School of Public Health, University of Colorado Anschutz Medical Campus, 13001 E. 17th Place, Aurora, CO 80045, United States; Applied Chemicals and Materials Division, National Institute of Standards and Technology, 325 Broadway Ave, Boulder, CO 80305, United States; Applied Chemicals and Materials Division, National Institute of Standards and Technology, 325 Broadway Ave, Boulder, CO 80305, United States

**Keywords:** breath, cannabis, forensic toxicology, Δ^9^-tetrahydrocannabinol (THC)

## Abstract

The increase of Δ^9^-tetrahydrocannabinol (THC) in breath after cannabis inhalation has been well-documented in the forensic field, but the trends after ingestion of cannabis-infused edibles have not yet been investigated. In this study, participants ingested a cannabis-infused edible and provided breath samples before and at three timepoints after ingestion. Participants were assigned to one of two breath sampling devices. THC was found in most pre-use breath samples, and THC concentration had variable trends after ingestion. Nineteen participants exhibited a maximum in their THC concentration at 47, 92, or 180 min after ingestion, while six participants had their highest THC concentration before the observed ingestion, and four participants had no significant change in THC concentration over the four samples. Five additional cannabinoids were detected in breath. While cannabidiol (CBD) trends followed THC trends for some participants, diverging trends in other participants suggest different biological processing of CBD derived from edibles. This proof-of-concept study shows that THC concentration in breath can increase after the ingestion of cannabis-infused edibles, but the uncertainty of breath measurements and a longer time window need to be further explored.

## Introduction

Currently there is no reliable measurement to determine if someone is driving under the influence of Δ^9^-tetrahydrocannabinol (THC), the main psychoactive component of cannabis [1]. Roadside screening devices are therefore currently focused on reliably identifying recent cannabis use [2]. Recent use is the determination that cannabis has been used within a certain timeframe (e.g., within a few hours of the test), instead of impairment from cannabis use. Oral fluid and breath are two noninvasive biological matrices that both have the advantage of being fully observable and practical for roadside collection. Roadside oral fluid collection for THC detection has been implemented or tested in some countries in Europe and North America. THC in oral fluid results from contamination of the oral cavity and a meta-analysis recommends against further widespread public testing due to high false positive rates [3]. Breath sampling has not been implemented to the same extent, but THC breath concentration has been monitored after known cannabis use with multiple devices [4–9]. Notably, there is evidence that breath samples can reflect systemic drugs from studies of drugs and/or their metabolites recovered from exhaled breath, such as methadone, heroin, tilidine, and tramadol [10, 11]. This provides a strong motivation to investigate breath as a biological matrix to readily identify recent cannabis use that is potentially independent of mode of consumption.

Quantitative breath-based measurements are well established for hydrogen, methane, and ethanol [12], with alcohol breathalyzers available for both law enforcement and personal use. However, THC quantitation in breath is more challenging than ethanol quantitation. THC is a large molecule that has a vapor pressure nine orders of magnitude lower than ethanol [13] and is expected to be found in the aerosol phase of breath. Therefore, researchers have focused on collecting breath aerosols with devices that employ electrostatic (interception) filters [4–6, 9, 14–16], impaction filters [7, 17], and devices that collect exhaled breath condensate [8]. Filter-based devices were designed to only collect exhaled breath aerosols, while condensate devices collect aerosols and condensed volatile compounds.

Breath studies have mainly focused on THC quantitation in breath after cannabis inhalation, despite the multiple modes of recreational cannabis consumption. One study quantified THC and cannabidiol (CBD) in breath after the use of an oral spray [9], but ingestion of cannabis as a digestible product has remained unexplored. The time course of intoxicating effects after edible ingestion is very different compared to inhalation. Additionally, THC can be detected in blood immediately after inhalation, while the ingestion of edibles leads to lower peak THC concentrations and a delayed peak onset [18, 19]. An increase in breath THC concentration after edible ingestion would be another step toward showing that breath-based THC measurements are a viable way to determine recent cannabis use with all modes of cannabis consumption.

The objective of this proof-of-concept study is to determine if THC, and potentially other cannabinoids, increase in breath after the ingestion of cannabis-infused edibles by sampling breath at three time points following cannabis ingestion. Two sampling devices were used. This study shows the first ever detection of cannabinoids in breath after the observed ingestion of cannabis-infused edibles.

## Materials and methods

### Study participants

The study described here is part of a larger study that aims to explore cannabis impairment assessments and driving performance in people using different cannabis products. The Colorado Multiple Institutional Review Board (COMIRB protocol 20-0949) and the National Institute of Standards and Technology (NIST protocol MML-2022-0396) approved study procedures. The research was conducted in accordance with the principles embodied in the Declaration of Helsinki and in accordance with local statutory requirements. All participants provided written informed consent that included consent to publish. Prior to data collection, participants were requested to abstain from ingesting cannabis-infused products for 12 h and from inhaling cannabis products for 8 h. Participants were advised to consume a light, low fat meal before their data collection visit. Participants brought their own cannabis-infused edible product (gummies) that had been procured from a licensed dispensary and labelled with THC quantity. Details about the packaging, product type, and cannabinoid dose were recorded. THC quantities consumed ranged from 5 to 100 mg, but label accuracy was not independently verified. On site, participants ingested their cannabis-infused edible and then waited 45 min to start post-use measurements. Participants were asked to abstain from consuming food or beverages, other than water, for at least 1 h post edible ingestion. As this is a proof-of-concept exploration into the detection of cannabinoids in breath after participants ingest edibles, statistical comparisons were not made between participants and their biometrics or previous cannabis product usage profiles.

### Breath sampling

Breath samples from 29 participants were collected at 20 ± 10 min (0.3 ± 0.2 h) before use (pre-use) and three timepoints after ingestion at 47 ± 4 min (0.79 ± 0.06 h), 92 ± 3 min (1.54 ± 0.04 h), and 180 ± 2 min (3.00 ± 0.08 h). Two different breath sampling devices were used. Twenty-three participants used an aerosol device (Breath Explor, Munkplast) at room temperature [17]. These participants have a “-B” after their participant id. A subset of participants (six total) used a condensate device (RTube, Respiratory Research) with a metal collar cooled to −80°C [8]. These participants have a “-R” after their participant id. The aerosol device contains three impaction filters that were analyzed separately and summed to determine cannabinoid masses on a per device basis. The condensate device contains one collection chamber. All participants followed a deep breathing pattern that increases the number of exhaled aerosols [20], which are hypothesized to carry the majority of THC molecules. For the aerosol device, the participant’s mouth contacts the mouthpiece only during exhalation so that no air is pulled through the device. For the condensate device, the participant’s mouth contacts the mouthpiece during inhalation and exhalation, as a two-way valve directs exhalations up into the collection chamber while inhalations pull air from the room. After each 5 min sampling period, devices were capped, placed in a plastic bag, and stored at −80°C until analysis.

### Breath analysis

Cannabinoids must be extracted by different methods for each device. The aerosol device contains three rigid filters that were analyzed separately following a microelution method [7]. After removing the filters from the device, 150 µL of a 35% H_2_O / 65% methanol solution (measured gravimetrically) with nominally 10 ng/g of each internal standard was added to the top of each filter. The filters were vortexed horizontally to get total solvent coverage and then were centrifuged to collect solvent at the bottom of the vial. Condensate samples were processed as described in Berry et al. [8]. Thawed condensate was removed from the collection chamber with a plunger that was cleaned between samples, re-frozen at −80°C, and then lyophilized to dryness in a vial covered by a lint-free wipe. Each participant’s complete set of samples was lyophilized together with a blank condensate sample (from a known noncannabis user) to check for transfer during processing. The lyophilized solids were reconstituted with 100 µL of 35% H_2_O / 65% methanol with nominally 10 ng/g of each internal standard.

Eleven cannabinoids were monitored by liquid chromatography–tandem mass spectrometry (LC–MS/MS): THC, Δ^8^-THC, Δ^10^-THC, CBD, cannabinol (CBN), cannabigerol (CBG), cannabichromene (CBC), tetrahydrocannabivarin (THCV), tetrahydrocannabinolic acid (THCA), cannabigerolic acid (CBGA), and 11-nor-9-carboxy-delta-9-tetrahydrocannabinol (THC-COOH). Each cannabinoid, except Δ^10^-THC, had a deuterated internal standard that was also monitored. Information on the separation and quantitation of these cannabinoids can be found in the Supplementary Data, including the method, quantitative accuracy (Supplemental Figure S1), interferences, and validation of microelution of the filters in the aerosol device (Supplemental Figure S2). Nine calibrators from 0.05 to 50 ng/g with a calibration curve weighting of 1/*x*^2^ were used to determine analyte concentration. Analytes were positively identified by their qualifier-to-quantifier ratios (±20%) and retention times compared to their respective deuterated internal standards (≤0.05 min). Concentrations were converted from ng/g to ng/device by multiplying by the elution solvent mass (aerosol device) or the reconstitution solvent mass (condensate device). As each filter from the aerosol device was analyzed separately, the final concentration was calculated by summing the detected masses from each filter. The variability of individual microeluted filters is roughly equal to the uncertainty of the analytical method (±20%) and was not biased by filter location (Supplemental Figure S3) or cannabinoid mass (Supplemental Figure S4), indicating the consistency of the microelution method. The lower limit of quantitation (LLOQ) for the analytical method, 0.04 ng/g, is equivalent to approximately 0.015 ng/device (0.005 ng/filter) or 0.003 ng/device for the aerosol device and condensate device, respectively. The limit of detection (LOD) was previously reported [8] and samples below LLOQ in which the cannabinoid was positively identified are designated trace.

## Results

### Cannabinoids detected in breath

**Figure 1.**
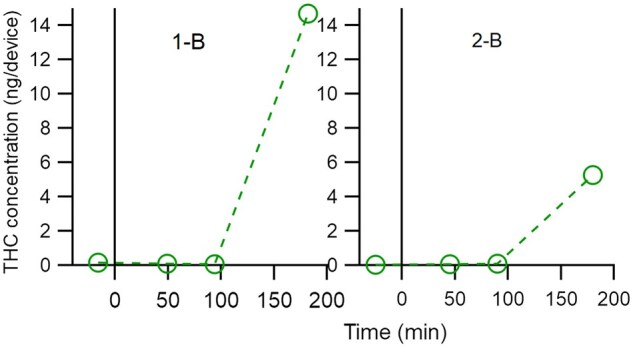
THC concentration (ng/device) in breath before and after ingestion of a cannabis-infused edible. The linear scale for THC concentration goes from 0 to 15 ng/device. Participants show a maximum THC concentration at 180 min after edible ingestion.

**Figure 2.**
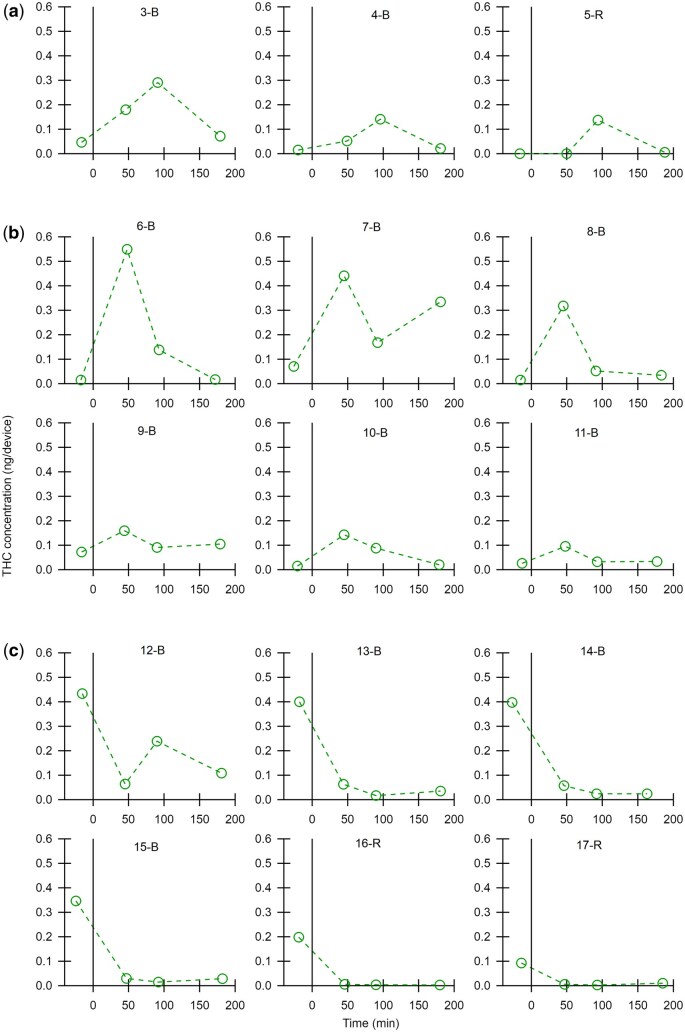
THC concentration (ng/device) in breath before and after ingestion of a cannabis-infused edible. The linear scale for THC concentration goes from 0 to 0.6 ng/device. Participants have different trends in THC concentration with (a) a maximum at 92 min after use, (b) a maximum at 47 min after use, and (c) a maximum THC concentration before ingestion.

**Figure 3.**
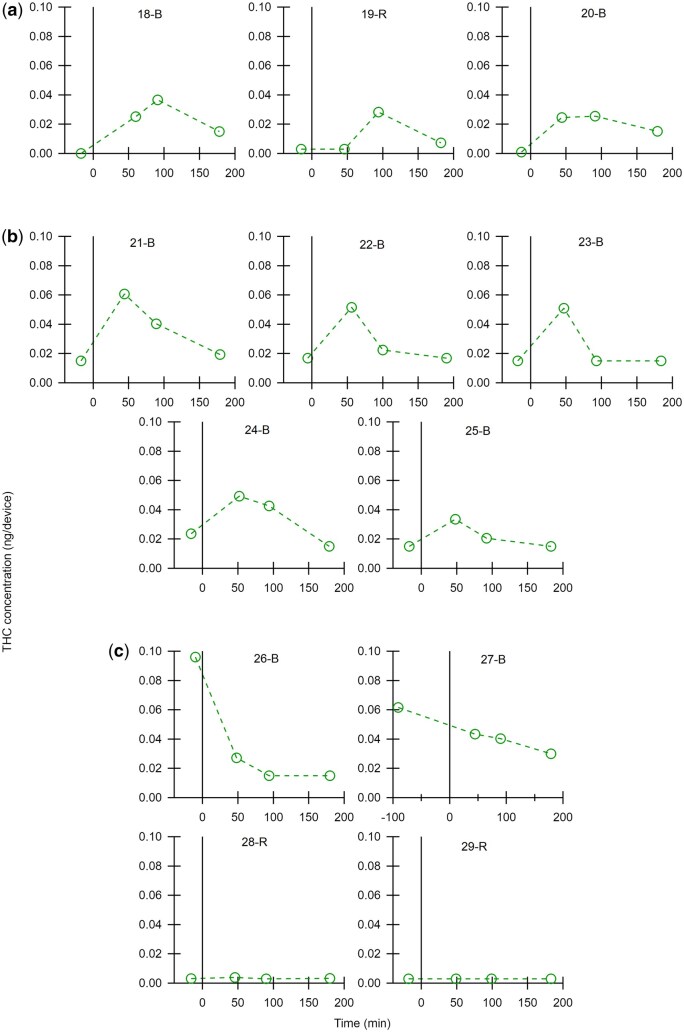
THC concentration (ng/device) in breath before and after ingestion of a cannabis-infused edible. The linear scale for THC concentration goes from 0 to 0.1 ng/device. Participants have different trends in THC concentration with (a) a maximum at 92 min after use, (b) a maximum at 47 min after use, and (c) a maximum THC concentration before ingestion.

THC was detected in most breath samples, both before and after cannabis-infused edible ingestion. THC was detected with both devices. While it was outside the scope of this study to directly compare devices, device types are specified within participant ids for transparency. In lieu of averages that may misrepresent the complexity of cannabinoid exhalation in breath following edible ingestion, all data is presented for each participant. THC concentration is shown graphically in three figures, sorted by trend in maximum THC concentration. Figure 1 displays participants that had a maximum THC concentration at the 180 min timepoint on a scale from zero to 15 ng/device. Figures 2 and 3 are grouped by maximum THC concentration timepoint (before or after ingestion) with Fig. 2 on a scale of zero to 0.6 ng/device and Fig. 3 on a scale of zero to 0.1 ng/device. Trace THC values in Figs 1–3 were set to the approximate LLOQ value for the device. No THC detected was set to zero. Tabulated data are also presented in Supplemental Table S2.

Six of the 29 participants had THC in their breath samples and no other cannabinoids that our method targeted. These participants had THC concentrations within the lowest range, presented in Fig. 3. The remaining 23 participants had at least one of the following five non-THC cannabinoids: CBD, CBN, CBG, CBC, and THCA. The concentrations of these cannabinoids can be found in Supplemental Table S2 and are graphically presented in Supplemental Figures S5–S7. CBD was detected in 11 participants, CBN was detected in 17 participants, and CBG was detected in 12 participants. These cannabinoids were detected with both devices. CBC was detected in four participants with the aerosol device. THCA was detected in one participant with the condensate device. No other cannabinoids from our method were detected.

**Figure 4.**
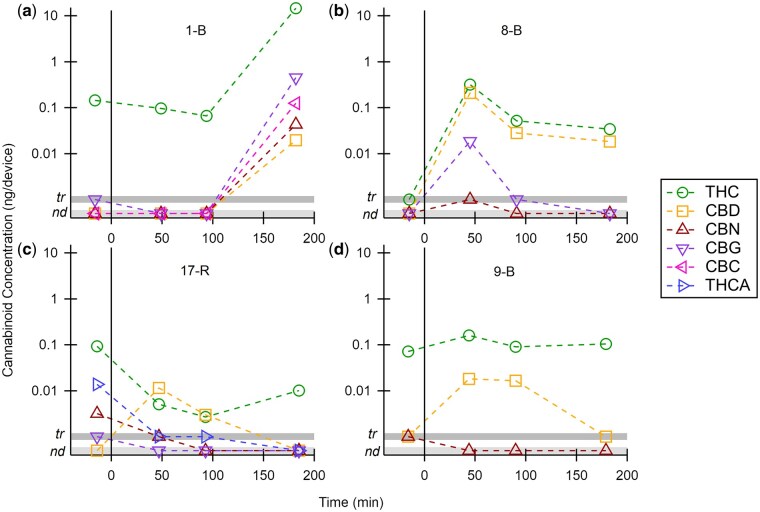
Cannabinoid concentration (ng/device) in breath before and after ingestion of a cannabis-infused edible on a log scale. Non-THC cannabinoid trends agree with THC trends for participants 1-B (a) and 8-B (b). CBD trends differ for participants 17-R (c) and 9-B (d), suggesting different processing for cannabinoids or high CBD edible consumption.

Figure 4 compares THC with other cannabinoids for select participants. For some participants, all cannabinoids follow the same trend, though their concentrations may differ by an order of magnitude or more. For example, for participant 1-B (Fig. 4a), the increase in THC at 180 min was matched by CBD, CBN, CBG, and CBC. For participant 8-B (Fig. 4b), the increase in THC at 47 min followed by the decrease at 92 min was matched by CBD and CBG. Five other participants (5-R, 10-B, 11-B, 24-B, 25-B) had similar trends. However, CBD trends did not always match THC trends. For participant 17-R (Fig. 4c), CBD increases at 47 min while THC decreases. At 180 min, the opposite occurs. For participant 9-B (Fig. 4d), THC and CBD both increase at 47 min, but CBD decreases at 180 min while THC remains constant. Four other participants (2-B, 4-B, 7-B, 27-B) had differing trends for CBD vs. THC.

## Discussion

### THC in breath before cannabis ingestion

THC was detected in breath samples from 27 of the 29 participants before edible ingestion, ranging from trace (10 participants) to greater than 0.4 ng/device. While participants were asked to abstain from cannabis use prior to the beginning of the data collection session, this phenomenon has been seen in many previous studies [4–8, 12, 15, 16]. Similar to the results from our study, some studies reported detecting THC in nearly 100% of pre-use samples [4, 5, 15]. Hubbard et al. [16] stands out as they reported THC detection in less than 5% of their study participants at pre-use. However, their analytical method had a LLOQ of 80 pg/device; only one of the breath samples in Fig. 3 had a THC concentration above this value. The LLOQ in our study was 15 pg/device or lower and the other studies that report ∼100% of participants with THC at pre-use had LLOQ that are ≤ 10 pg/device. Additionally, Hubbard et al. [16] only sampled breath for ∼3 min, while our study sampled for 5 min, thereby concentrating THC from a larger breath volume. The finding of detectable and/or quantifiable THC before cannabis use shows how challenging it is to interpret a single THC concentration measurement in breath with no prior information about THC at baseline.

### THC kinetics in breath after ingestion of cannabis-infused edibles

The detection of THC in breath after the ingestion of cannabis-infused edibles has been a large knowledge gap in the field of forensic breath sampling. The results presented in Figs 1–3 show that THC trends after the ingestion of edibles vary greatly between participants. Figures 1, 2a, 2b, and 3a, 3b show that edible ingestion can increase the quantity of THC in exhaled breath within 180 min or less.

However, Figs 2c and 3c show a decrease in THC concentration after edible ingestion and can be interpreted in different ways. One potential explanation is that the study length was not long enough to detect the post-use increase in THC concentration. Reviews of pharmacokinetic data after oral ingestion found that maximum plasma THC concentrations are observed anywhere from less than an hour to 4 h post use [18], suggesting that for some participants in this study the peak THC concentration could be outside the 3 h study window. Additionally, breath sample quality could have impacted the detected THC concentrations. Breath is a novel matrix, and the human factors and sampling factors that influence breath THC concentration have not been fully identified. While each participant provided four breath samples nominally following the same sampling protocol, the sampling time (5 min) provides only limited evidence of consistency. The different peak concentration times for participants could have been impacted by different digestion patterns between participants or other biometric data not explored here.

**Figure 5.**
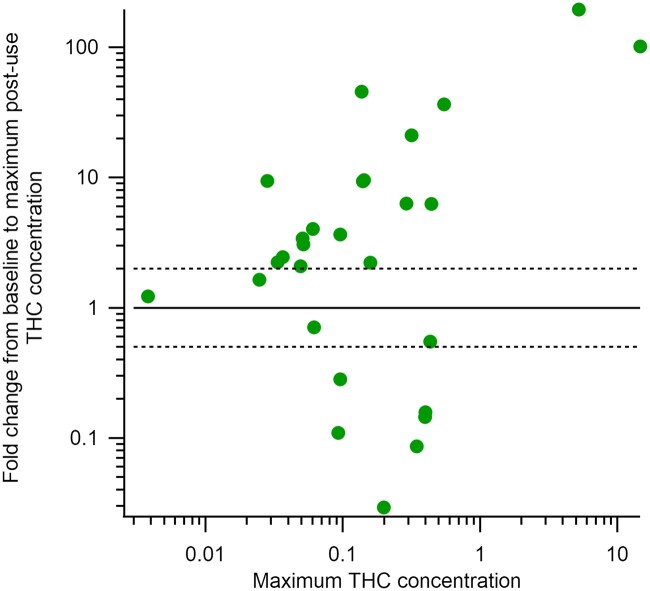
Fold change in THC concentration in breath from pre-use to maximum post-use concentration compared to the maximum THC concentration for each participant. Points above the solid line signify an increase in THC concentration after cannabis ingestion, while points below the solid line signify a decrease after cannabis ingestion. The dashed lines signify our estimated significance threshold.

To begin considering whether observed changes in THC concentration can be judged significant, we examined the fold change in THC concentration after edible ingestion (Fig. 5). This figure displays the fold change from pre-use THC concentration to the maximum THC concentration after cannabis ingestion as a function of the maximum THC concentration detected from all timepoints for each participant. While the analytical method uncertainty is within ± 20%, there will also be unknown uncertainties associated with breath volume, breath velocity, aerosol production, and cannabinoid recovery. Therefore, we set the criteria for a significant fold change to greater than a double or halving of the pre-use concentration. This is designated in Fig. 5 with the dashed lines at fold changes of 2 and 0.5. Figure 5 shows that fold changes do not seem to be related to the maximum THC concentration detected in breath. For participants with no THC or a trace THC concentration in their pre-use sample, we set the THC concentration to their device’s respective LLOQ so that a noninfinite fold change could be calculated. The participant with consistent trace concentrations across all samples (29-R) was not included in this figure.

Figure 5 shows that all participants, except for four, have a significant change in THC concentration after cannabis ingestion. However, both significant increases (19 participants) and significant decreases (6 participants) were observed. As participants were monitored during ingestion, the significant increases are assumed to be from the cannabis-infused edible ingestion. The six participants with significant decreases had high absolute THC concentrations before cannabis ingestion. Only two other participants (1-B and 12-B) had a pre-use value of this magnitude. Perhaps not surprisingly, THC concentrations for these six participants decreased at 47 min by a factor of three or more, calling into question whether their pre-use THC concentration truly represents a baseline value. By definition, baseline values should not change, but no one has ever made multiple measurements prior to cannabis use to determine the uncertainty in these measurements or the timeframe over which they can be considered stable.

Despite the variability in these results, an increased THC concentration in breath can be detected after ingesting edibles. More research is necessary with statistically significant sample populations to understand the pharmacokinetics of THC elimination in exhaled breath and the absorption and distribution of THC throughout the body. Future breath studies should collect breath from participants for a longer time after ingestion and collect more frequently to ensure the peak concentration is not missed.

### Kinetics of non-THC cannabinoids

Previous studies have suggested that the detection of non-THC cannabinoids could be useful in determining recent use. For example, in whole blood, molar metabolite ratios utilizing THC metabolites were better indicators than THC alone [21]. Here, non-THC cannabinoids were detected in 23 participants, and 13 had at least three non-THC cannabinoids. CBN and CBG trends matched THC trends, as exemplified by participants 1-B and 8-B (Fig. 4a, 4b) and by other participants (e.g. 2-B and 5-R). CBC and THCA were less commonly detected but also followed these trends. These results suggest that the presence of non-THC cannabinoids in these breath samples comes from the same recent edible ingestion. CBD trends are more difficult to explain. For example, THC and CBN decrease after ingestion for participant 17-R (Fig. 4c), but CBD increases, perhaps due to a higher CBD content in their edible or undisclosed CBD usage. Participant 9-B (Fig. 4d) represents several participants for whom CBD trends match THC trends at some timepoints and deviate at other timepoints (e.g. 2-B and 4-B). Although the observed trends are interesting, this was not the focus of our study.

### Limitations

This study has a number of limitations. Future studies on cannabinoids in breath following edible ingestion should include a larger number of participants using more than one collection device with a robust statistical analysis. If possible, future studies should confirm abstinence within a closed research unit and standardize the dosing protocol. In our study design, participants provided three breath samples up to approximately three hours after ingestion and for some individuals the level of THC in the breath may not have peaked; thus, future studies should include more frequent and additional later time points to ensure the peak is not missed. More research is underway to investigate breath sample quality and other factors that could impact the detected cannabinoid concentrations, as breath velocity [22], aerosol emissions in breath [23], and digestive system contents may impact breath collection. While our study did not seek to correlate THC breath concentration to THC dose and impairment, this is also an extremely important area of consideration for future research.

## Conclusions

In this proof-of-concept study, participants ingested cannabis-infused edibles and provided breath samples before and after cannabis ingestion with devices designed to collect exhaled breath aerosols or exhaled breath condensate. THC was detected in all breath samples after cannabis use with both devices, although with variable trends. When detected, CBN and CBG matched THC trends, but there were multiple instances where CBD and THC disagreed, suggesting differences in biological processing or clearance. Of the 29 participants in this study, 19 showed a significant increase in THC concentration after edible ingestion (at any of the three post-use timepoints), 4 showed no change, and 6 showed a significant decrease in THC concentration after edible use. Those with significant decreases had some of the highest pre-use THC concentrations and had a factor of 3 or more decrease from pre-use to their first post-use THC measurement. This observation highlights the need for repeat measurements at each participant’s presumed baseline. Breath samples from two-thirds of the participants in this study showed an increase in THC concentration after cannabis ingestion, but the remaining one-third are not necessarily inconsistent with the hypothesis that recent cannabis use can be detected in breath, regardless of the mode of use. The post-use timepoints in this study were limited, with a gap between 92 and 180 min and no samples after 180 min. Therefore, the peak THC concentration in one or more participants may have been missed. Overall, this study provides additional support to the hypothesis that breath-based measurements could be used to determine recent cannabis use despite multiple modes of consumption. However, the high detection rate of THC in breath after 8 h of requested abstinence shows that identifying recent use from a single measurement is challenging from any matrix. Future work will focus on determining if THC’s rate-of-change between two breath samples is a better indication of recent use than THC concentration in a single breath sample.

## Supplementary Material

bkaf063_Supplementary_Data

## Data Availability

The data underlying this article will be shared on reasonable request to the corresponding author.
