# Correction to: The detection of cannabinoids in breath after ingestion of cannabis-infused edibles

**DOI:** 10.1093/jat/bkag018

**Published:** 2026-05-25

This is a correction to: Jennifer L Berry, Ashley Brooks-Russell, Tara M Lovestead, Kavita M Jeerage, The detection of cannabinoids in breath after ingestion of cannabis-infused edibles, *Journal of Analytical Toxicology*, Volume 49, Issue 9, November 2025, Pages 673–680, https://doi.org/10.1093/jat/bkaf063

The last name of the first author is emended to read: “Jennifer L Berry”.

