# Supplementary material for: The detection of cannabinoids in breath after ingestion of cannabis-infused edibles
Source: J Anal Toxicol. 2025 Jul 10;49(9):673–80. doi: 10.1093/jat/bkaf063 (PMC12842605; doi:10.1093/jat/bkaf063)
Supplement: bkaf063_Supplementary_Data [file bkaf063_supplementary_data.docx]

**Supplementary Data**

**Materials**

Standards and deuterated internal standards (designated by -d3) were purchased commercially and used as received for Δ^9^-tetrahydrocannabinol (THC), Δ^8^-tetrahydrocannabinol (Δ^8^-THC), Δ^10^-tetrahydrocannabinol (Δ^10^-THC), cannabidiol (CBD), cannabinol (CBN), cannabigerol (CBG), cannabichromene (CBC), tetraydrocannabivarin (THCV), tetrahydrocannabinolic acid (THCA), cannabigerolic acid (CBGA), and 11-nor-9-carboxy-delta-9-tetrahydrocannabinol (THC-COOH). Deuterated internal standards were available for all analytes, except for Δ^10^-THC where the deuterated standard was not available at the time. Liquid chromatography-mass spectrometry grade solvents were purchased commercially and used as received. Stock solutions used to create calibration curves, reconstitute lyophilized condensate samples, and elute impaction filters were made in methanol, stored at -80 °C, and used within 60 days. Solvent from reconstitution of lyophilized condensate samples and solvent from microelution of the impaction filters were transferred to silanized glass inserts in autosampler vials for analysis. Analytical standards used for the calibration curve were analyzed in silanized autosampler vials.

**HPLC-MS/MS**

An Agilent 1290 Infinity II high performance liquid chromatography instrument with a 6470 tandem quadrupole mass spectrometer (LC-MS/MS) was used. All information can be found in Berry et al. [1], so only a short description follows. The analytical column (ZORBAX RRHD Eclipse Plus C18, 2.1 x 50 mm, 1.8 µm particle size) was preceded by a 5 mm guard column with the same stationary phase. Solvent A was 0.1% formic acid in water (H_2_O) and solvent B was 0.1% formic acid in methanol. System suitability checks were performed before each experimental run with an isocratic method (10% A / 90% B) with 20 µL injections of a ~1 ng/g mixture of THC and THC-d3 in 10% A / 90% B. The gradient method used to separate the 11 cannabinoids and 10 deuterated cannabinoids had a total run time of 11 min. The gradient method is as follows: starting conditions of 65% B, increase to 70% B at 5 min, 80% B at 7 min, 85% B at 8 min with a 1.5 min hold, followed by a 1.5 min reset to starting conditions. The flow rate was 0.7 mL/min, the column temperature was 50 °C, the injection volume was 20 µL, and the autosampler tray temperature was 4 °C.

The dynamic multiple reaction monitoring (d-MRM) window was ±0.5 min of the peak retention time for each cannabinoid (Table S1). The deuterated internal standards elute <0.05 min before the standards. No deuterated internal standard was available for Δ^10^-THC, so Δ^8^-THC-d3 was used for Δ^10^-THC quantitation because it was closest in retention time and structure. The MS/MS source parameters included a sheath gas temperature of 375 °C, sheath gas flow of 12 L/min, nebulizer pressure of 45 psi, positive and negative nozzle voltage of 2000 V, gas temperature of 300 °C, gas flow of 12 L/min, positive and negative capillary voltage of 3500 V, and a positive and negative delta electron multiplier voltage (ΔEMV) of 500 V. Table S1 also lists the ionization modes, precursor and product ions, fragmentor and collision energies, and product ion ratios for each cannabinoid and its deuterated internal standard. The product ion ratios were set based on each batch’s calibrators, while the values listed in Table S1 are averages from all batches.

Table S1. Retention time and mass spectrometry parameters for each cannabinoid’s precursor, quantifier (Q), and qualifier (q) ions.

| **Cannabinoid** | **Retention Time (min)** | **Mode** | **Precursor (m/z)** | **Q**  **(m/z)** | **Q Fragmentor Energy (V)** | **Q**  **Collision Energy (V)** | **q**  **(m/z)** | **q Fragmentor Energy (V)** | **q**  **Collision Energy (V)** | **q/Q Ratio** |
| --- | --- | --- | --- | --- | --- | --- | --- | --- | --- | --- |
| THC-COOH | 3.75 | Neg | 343.2 | 299.2 | 156 | 24 | 245.1 | 156 | 32 | 22 |
| THCV | 4.00 | Pos | 287.2 | 165.1 | 109 | 22 | 123.0 | 109 | 38 | 42 |
| CBD | 4.30 | Pos | 315.2 | 193.1 | 116 | 24 | 123.0 | 121 | 40 | 60 |
| CBG | 4.58 | Pos | 317.2 | 193.1 | 97 | 18 | 123.0 | 97 | 38 | 27 |
| CBGA | 6.13 | Neg | 359.2 | 341.2 | 136 | 22 | 315.3 | 136 | 22 | 38 |
| CBN | 6.31 | Pos | 311.2 | 223.1 | 120 | 23 | 178.0 | 120 | 75 | 27 |
| THC | 6.98 | Pos | 315.2 | 193.1 | 136 | 24 | 123.0 | 136 | 36 | 60 |
| Δ^8^-THC | 7.21 | Pos | 315.2 | 193.1 | 122 | 24 | 123.0 | 122 | 40 | 65 |
| Δ^10^-THC | 7.57 | Pos | 315.2 | 193.1 | 140 | 24 | 123.0 | 133 | 38 | 51 |
| CBC | 7.79 | Pos | 315.2 | 193.1 | 102 | 14 | 259.2 | 102 | 18 | 53 |
| THCA | 8.51 | Neg | 357.2 | 313.2 | 141 | 26 | 245.1 | 141 | 34 | 19 |
| DEUTERATED INTERNAL STANDARDS | | | | | | | | | | |
| THC-COOH-d3 | 3.72 | Neg | 346.2 | 302.2 | 140 | 24 | 248.1 | 140 | 32 | 20 |
| THCV-d3 | 3.97 | Pos | 290.2 | 168.1 | 109 | 22 | 123.0 | 109 | 38 | 41 |
| CBD-d3 | 4.27 | Pos | 318.2 | 196.1 | 106 | 24 | 123.0 | 106 | 36 | 60 |
| CBG-d3 | 4.55 | Pos | 320.3 | 196.1 | 97 | 14 | 123.0 | 97 | 38 | 29 |
| CBGA-d3 | 6.10 | Neg | 362.2 | 344.3 | 126 | 22 | 318.3 | 126 | 22 | 38 |
| CBN-d3 | 6.29 | Pos | 314.2 | 223.1 | 125 | 23 | 178.0 | 125 | 79 | 28 |
| THC-d3 | 6.95 | Pos | 318.2 | 196.1 | 116 | 24 | 123.0 | 116 | 40 | 62 |
| Δ^8^-THC-d3 | 7.19 | Pos | 318.2 | 196.1 | 127 | 24 | 123.1 | 127 | 36 | 65 |
| CBC-d3 | 7.77 | Pos | 318.2 | 196.1 | 97 | 14 | 262.2 | 97 | 18 | 56 |
| THCA-d3 | 8.51 | Neg | 360.2 | 316.3 | 145 | 26 | 248.2 | 145 | 38 | 20 |

**Validation of Cannabinoid Microelution with the Aerosol Device**

The analytical method described by Berry et al. [1] utilized calibrators with nominal cannabinoid concentrations from 0.04 ng/g to 250 ng/g. Here, a smaller range was utilized to better match the cannabinoid concentrations found in authentic samples. Nominal concentrations were L1 ≈ 50 ng/g, L2 ≈ 10 ng/g, L3 ≈ 5 ng/g, L4 ≈ 2.5 ng/g, L5 ≈ 1 ng/g, L6 ≈ 0.5 ng/g, L7 ≈ 0.25 ng/g, L8 ≈ 0.1 ng/g, and L9 ≈ 0.05 ng/g, with internal standard concentrations of approximately 10 ng/g. Figure S1 shows that calibrator accuracy was within 20% for 10 of the 11 cannabinoids investigated in this study (Δ^10^-THC was an exception).


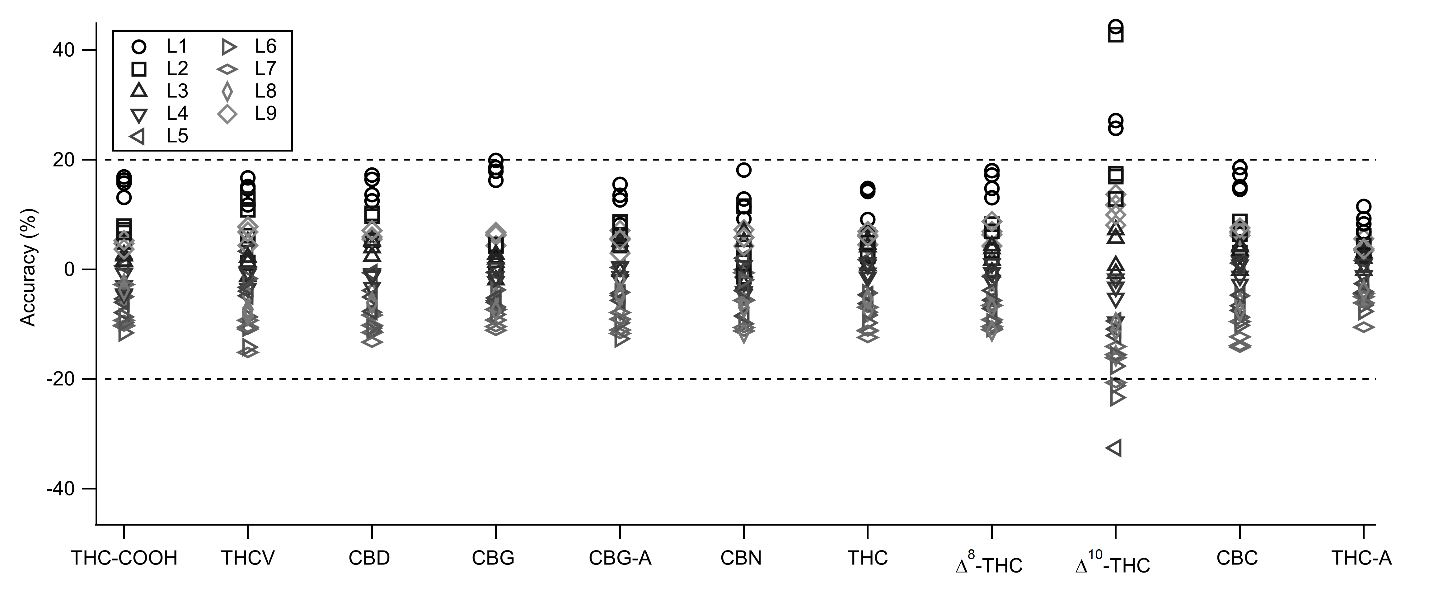


Figure S1. Accuracy of calibrators prepared on four different days from two batches of stock solutions. The calibration curves of relative response vs. concentration utilized 1/x^2^ weighting. Calculated concentrations determined from the average of three injections were compared to the gravimetric concentrations to calculate accuracy.

The microelution method described by Henion et al. [2] involves adding elution solvent containing internal standards to each impaction filter, vortexing so the solvent contacts all filter surfaces, and centrifuging to recover the solvent. No evaporative concentration is involved, so the mass of elution solvent directly affects analyte concentration in the resulting samples. We determined this mass gravimetrically after adding 150 µL of elution solvent (starting conditions of our LC mobile phase) with a positive displacement pipette. Impaction filters from unused devices and devices with blank breath matrix from known non-users were processed by the microelution method and analyzed as described above. Consistent with other control experiments, there was no signal from the deuterated internal standards or the solvent that matched the retention time and monitored transitions of the 11 cannabinoids investigated in this study, with the exception of a small THCV peak from lower purity THCV-d3 (data not shown). The unused devices in these experiments had a peak that overlapped with the Δ^8^-THC qualifier transition (≤ 0.06 min apart) and did not allow for accurate determination of the q/Q ratio (data not shown). The unused devices also had a peak that overlapped with the THCA quantifier transition, but this peak was ≥ 0.15 min apart and accurate q/Q ratio determination was still possible (data not shown). Blank breath matrix did not show any additional interferences. Therefore, based on calibrator accuracy and potential interference, Δ^10^-THC, THCV, and Δ^8^-THC would be treated with caution if detected in authentic samples.

Cannabinoid recovery from the impaction filter material was investigated by comparing eight quality control (QC) samples prepared with cannabinoids (0.04 ng/g) and internal standards (10 ng/g) at the lower limit of quantitation (LLOQ). Four of the samples were analyzed immediately after preparation, while the other four samples were added to filters with blank breath matrix from known non-users. These filters were then processed following the microeleution method, that is, they were vortexed horizontally for 5 min and centrifuged for 5 min to recover the solvent. Figure S2 shows that QC accuracy was within 20% for 10 of the 11 cannabinoids investigated in this study (Δ^10^-THC was an exception, as expected). Nine additional QC samples prepared with cannabinoids (three samples at 1 ng/g, three samples at 10 ng/g, and three samples at 50 ng/g) and internal standards (10 ng/g) were similarly added to filters with blank breath matrix. For these higher spiked masses, QC accuracy was within 30% for Δ^10^-THC and within 20% for all other cannabinoids investigated in this study.


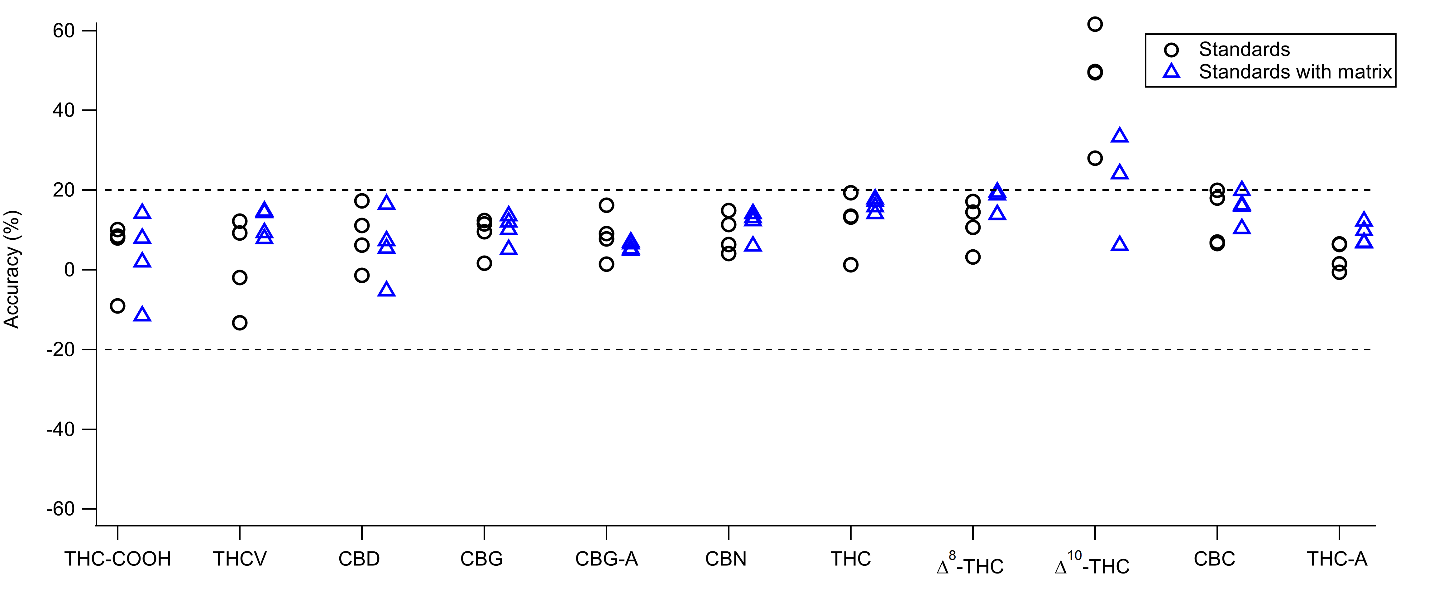


Figure S2. Accuracy of QC samples prepared at 0.04 ng/g from two batches of stock solutions. Calculated concentrations determined from the average of three injections were compared to the gravimetric concentrations to calculate accuracy. QCs labeled “standards” were prepared gravimetrically on four days (one QC each day) and analyzed after preparation. QCs labeled “standards with matrix” were prepared gravimetrically on four different days (again, one QC each day) and added to impaction filters with blank breath matrix.

**Variability of Cannabinoids Recovered from Individual Filters**

The aerosol device used in this study contains three impaction filters and each filter was analyzed individually prior to being summed to determine the detected cannabinoid mass per device. To investigate variability, the difference of each filter from the average for the device was calculated for each device that had cannabinoid concentrations greater than the LLOQ for all filters. Figure S3 shows that most filters (89% for THC, 85% for CBD, 78% for CBN and CBC, and 100% for CBG) varied from the device average by 20% or less, with the remaining filters within 50%. For comparison, the accuracy of the analytical method is ± 20%. Additionally, we do not observe a bias between the middle filter and the two side filters. Figure S4 shows that the cannabinoid mass detected in a filter did not influence the difference of the filter from the device average. This remains true for the devices with the highest cannabinoid masses, from participants 1-B (two of the three filters overlap) and 2-B, providing evidence that these samples were not anomalous. These results demonstrate that the variability of microeluted filters is roughly equal to the variability of the analytical method.


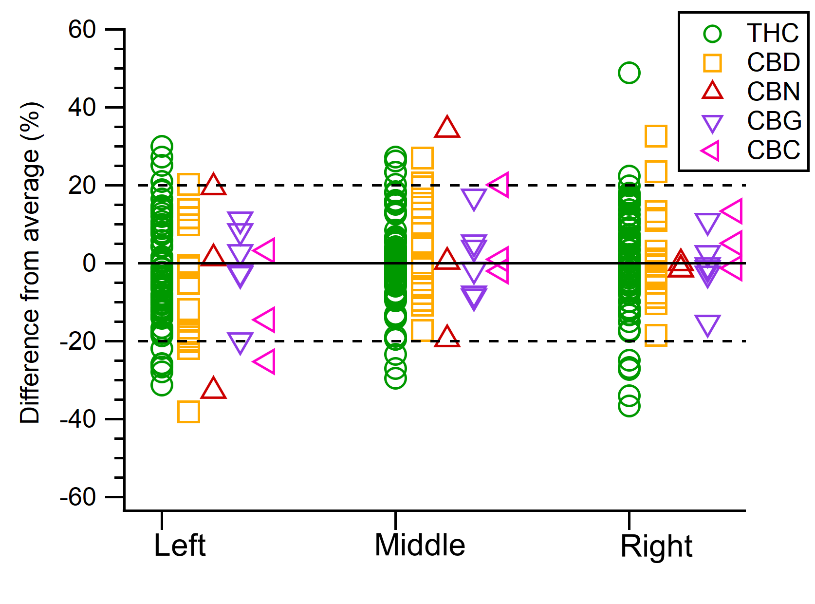


Figure S3. The difference of the detected cannabinoid mass in each filter from the average for the device shown as a function of filter position. This calculation was only done when there were detected concentrations above the LLOQ for every filter in the device.


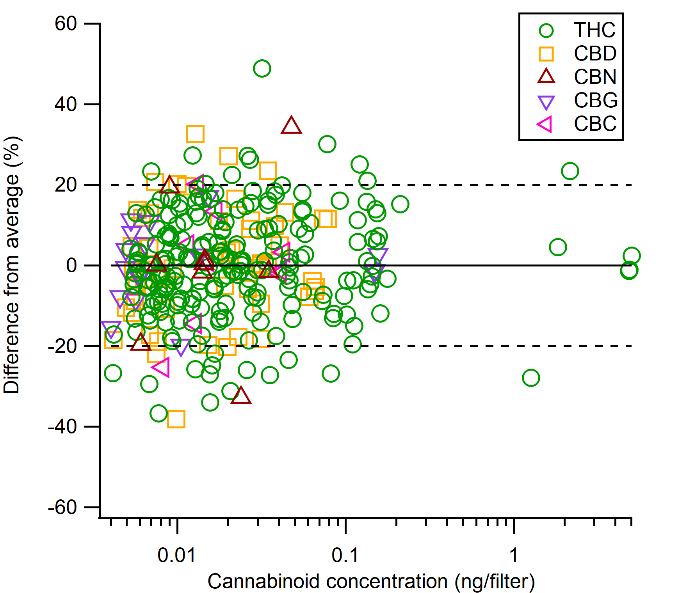


Figure S4. The difference of the detected cannabinoid mass in each filter from the average for the device shown as a function of the cannabinoid concentration for that filter. This calculation was only done when there were detected concentrations above the LLOQ for every filter in the device.

**Cannabinoid Concentrations in Breath Samples**

Table S2. Detected concentrations of cannabinoids from all participants with two different collection devices (B or R) in ng/device. When the detected cannabinoid concentration is below the LLOQ (0.04 ng/g) but still passes the other identification requirements, the concentration is designated by trace (tr).

|  | | **Pre-use** | **Post #1** | **Post #2** | **Post #3** |
| --- | --- | --- | --- | --- | --- |
| **1-B** | THC | 0.144 | 0.097 | 0.066 | 14.697 |
|  | CBD | — | — | — | 0.020 |
|  | CBN | — | — | — | 0.043 |
|  | CBG | *tr* | — | — | 0.453 |
|  | CBC | — | — | — | 0.125 |
|  | THCA | — | — | — | — |
| **2-B** | THC | 0.027 | 0.064 | 0.083 | 5.252 |
|  | CBD | — | 0.116 | 0.083 | 0.057 |
|  | CBN | — | *tr* | *tr* | 0.107 |
|  | CBG | — | *tr* | *tr* | 0.039 |
|  | CBC | — | — | — | 0.033 |
|  | THCA | — | — | — | — |
| **3-B** | THC | 0.046 | 0.179 | 0.291 | 0.071 |
|  | CBD | — | *tr* | *tr* | — |
|  | CBN | — | *tr* | *tr* | — |
|  | CBG | — | — | — | — |
|  | CBC | — | — | — | — |
|  | THCA | — | — | — | — |
| **4-B** | THC | *tr* | 0.052 | 0.140 | 0.021 |
|  | CBD | — | 0.025 | *tr* | *tr* |
|  | CBN | — | — | — | — |
|  | CBG | — | — | — | — |
|  | CBC | — | — | — | — |
|  | THCA | — | — | — | — |
| **5-R** | THC | — | — | 0.137 | 0.005 |
|  | CBD | — | — | — | — |
|  | CBN | — | — | 0.004 | — |
|  | CBG | — | — | 0.005 | — |
|  | CBC | — | — | — | — |
|  | THCA | — | — | — | — |
| **6-B** | THC | *tr* | 0.549 | 0.138 | 0.017 |
|  | CBD | — | — | — | — |
|  | CBN | — | *tr* | — | — |
|  | CBG | — | *tr* | — | — |
|  | CBC | — | — | — | — |
|  | THCA | — | — | — | — |
| **7-B** | THC | 0.070 | 0.441 | 0.167 | 0.34 |
|  | CBD | — | 0.198 | 0.094 | 0.015 |
|  | CBN | *tr* | *tr* | *tr* | *tr* |
|  | CBG | — | *tr* | — | *tr* |
|  | CBC | — | — | — | *tr* |
|  | THCA | — | — | — | — |
| **8-B** | THC | *tr* | 0.318 | 0.052 | 0.034 |
|  | CBD | — | 0.210 | 0.028 | 0.018 |
|  | CBN | — | *tr* | — | — |
|  | CBG | — | 0.018 | *tr* | — |
|  | CBC | — | — | — | — |
|  | THCA | — | — | — | — |
| **9-B** | THC | 0.072 | 0.159 | 0.091 | 0.105 |
|  | CBD | *tr* | 0.018 | 0.017 | *tr* |
|  | CBN | *tr* | — | — | — |
|  | CBG | — | — | — | — |
|  | CBC | — | — | — | — |
|  | THCA | — | — | — | — |
| **10-B** | THC | *tr* | 0.143 | 0.088 | 0.020 |
|  | CBD | — | — | — | — |
|  | CBN | — | — | — | — |
|  | CBG | — | 0.015 | *tr* | — |
|  | CBC | — | — | — | — |
|  | THCA | — | — | — | — |
| **11-B** | THC | 0.026 | 0.096 | 0.033 | 0.034 |
|  | CBD | — | — | — | — |
|  | CBN | — | — | — | — |
|  | CBG | — | — | — | — |
|  | CBC | — | 0.045 | *tr* | — |
|  | THCA | — | — | — | — |
| **12-B** | THC | 0.433 | 0.064 | 0.238 | 0.109 |
|  | CBD | — | — | — | — |
|  | CBN | *tr* | *tr* | *tr* | *tr* |
|  | CBG | 0.018 | — | *tr* | — |
|  | CBC | — | — | — | — |
|  | THCA | — | — | — | — |
| **13-B** | THC | 0.400 | 0.063 | 0.017 | 0.035 |
|  | CBD | — | — | — | — |
|  | CBN | 0.023 | *tr* | *tr* | *tr* |
|  | CBG | 0.015 | — | — | — |
|  | CBC | — | — | — | — |
|  | THCA | — | — | — | — |
| **14-B** | THC | 0.397 | 0.057 | 0.024 | 0.024 |
|  | CBD | — | — | — | — |
|  | CBN | *tr* | — | — | — |
|  | CBG | *tr* | — | — | — |
|  | CBC | — | — | — | — |
|  | THCA | — | — | — | — |
| **15-B** | THC | 0.346 | 0.030 | *tr* | 0.029 |
|  | CBD | — | — | — | — |
|  | CBN | *tr* | — | — | — |
|  | CBG | *tr* | — | — | — |
|  | CBC | — | — | — | — |
|  | THCA | — | — | — | — |
| **16-R** | THC | 0.199 | 0.006 | 0.003 | *tr* |
|  | CBD | — | — | — | — |
|  | CBN | 0.010 | *tr* | *tr* | — |
|  | CBG | 0.006 | — | — | — |
|  | CBC | — | — | — | — |
|  | THCA | — | — | — | — |
| **17-R** | THC | 0.093 | 0.005 | 0.003 | 0.010 |
|  | CBD | — | 0.012 | 0.003 | — |
|  | CBN | 0.003 | *tr* | — | — |
|  | CBG | *tr* | — | — | — |
|  | CBC | — | — | — | — |
|  | THCA | 0.014 | *tr* | *tr* | — |
| **18-B** | THC | — | 0.025 | 0.037 | *tr* |
|  | CBD | — | — | — | — |
|  | CBN | — | — | *tr* | — |
|  | CBG | — | — | — | — |
|  | CBC | — | — | — | — |
|  | THCA | — | — | — | — |
| **19-R** | THC | *tr* | *tr* | 0.028 | 0.007 |
|  | CBD | — | — | — | — |
|  | CBN | — | — | *tr* | — |
|  | CBG | — | — | — | — |
|  | CBC | — | — | — | — |
|  | THCA | — | — | — | — |
| **20-B** | THC | *tr* | 0.025 | 0.026 | 0.015 |
|  | CBD | — | — | — | — |
|  | CBN | — | — | — | — |
|  | CBG | — | — | — | — |
|  | CBC | — | — | — | — |
|  | THCA | — | — | — | — |
| **21-B** | THC | *tr* | 0.061 | 0.040 | 0.019 |
|  | CBD | — | — | — | — |
|  | CBN | — | — | — | — |
|  | CBG | — | — | — | — |
|  | CBC | — | — | — | — |
|  | THCA | — | — | — | — |
| **22-B** | THC | 0.017 | 0.052 | 0.022 | 0.017 |
|  | CBD | — | — | — | — |
|  | CBN | — | — | — | — |
|  | CBG | — | — | — | — |
|  | CBC | — | — | — | — |
|  | THCA | — | — | — | — |
| **23-B** | THC | *tr* | 0.051 | *tr* | *tr* |
|  | CBD | — | — | — | — |
|  | CBN | — | — | — | — |
|  | CBG | — | — | — | — |
|  | CBC | — | — | — | — |
|  | THCA | — | — | — | — |
| **24-B** | THC | 0.024 | 0.049 | 0.043 | *tr* |
|  | CBD | — | 0.104 | 0.061 | *tr* |
|  | CBN | — | — | — | — |
|  | CBG | — | — | — | — |
|  | CBC | — | — | — | — |
|  | THCA | — | — | — | — |
| **25-B** | THC | *tr* | 0.034 | 0.020 | *tr* |
|  | CBD | — | 0.074 | 0.029 | — |
|  | CBN | — | — | — | — |
|  | CBG | — | — | — | — |
|  | CBC | — | — | — | — |
|  | THCA | — | — | — | — |
| **26-B** | THC | 0.096 | 0.027 | *tr* | *tr* |
|  | CBD | — | — | — | — |
|  | CBN | — | — | — | — |
|  | CBG | — | — | — | — |
|  | CBC | — | — | — | — |
|  | THCA | — | — | — | — |
| **27-B** | THC | 0.062 | 0.043 | 0.040 | 0.030 |
|  | CBD | 0.048 | *tr* | — | — |
|  | CBN | — | — | — | — |
|  | CBG | — | — | — | — |
|  | CBC | — | — | — | — |
|  | THCA | — | — | — | — |
| **28-R** | THC | 0.003 | 0.004 | 0.003 | 0.003 |
|  | CBD | — | — | — | — |
|  | CBN | — | — | — | — |
|  | CBG | — | — | — | — |
|  | CBC | — | — | — | — |
|  | THCA | — | — | — | — |
| **29-R** | THC | *tr* | *tr* | *tr* | *tr* |
|  | CBD | — | — | — | — |
|  | CBN | — | — | — | *tr* |
|  | CBG | — | — | — | — |
|  | CBC | — | — | — | — |
|  | THCA | — | — | — | — |


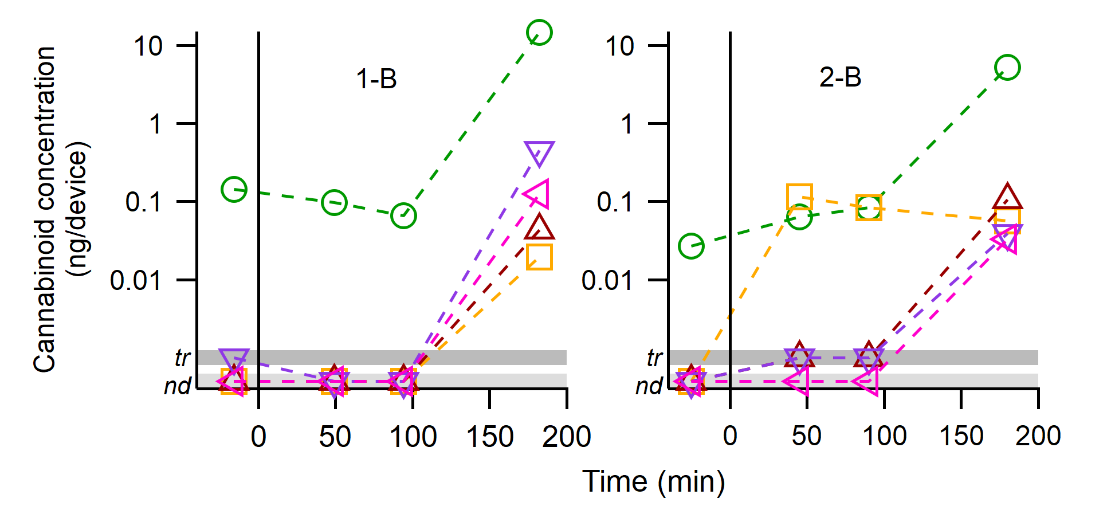


Figure S5. Cannabinoid concentration (ng/device) in breath before and after ingestion of a cannabis-infused edible on a log scale. Participants show a maximum THC concentration at 180 min after edible ingestion. These participants are the same as shown in Figure 1 in the main text. THC are green circles, CBD are yellow squares, CBN are maroon upward-pointing triangles, CBG are purple downward-pointing triangles, and CBC are pink leftward-pointing triangles.


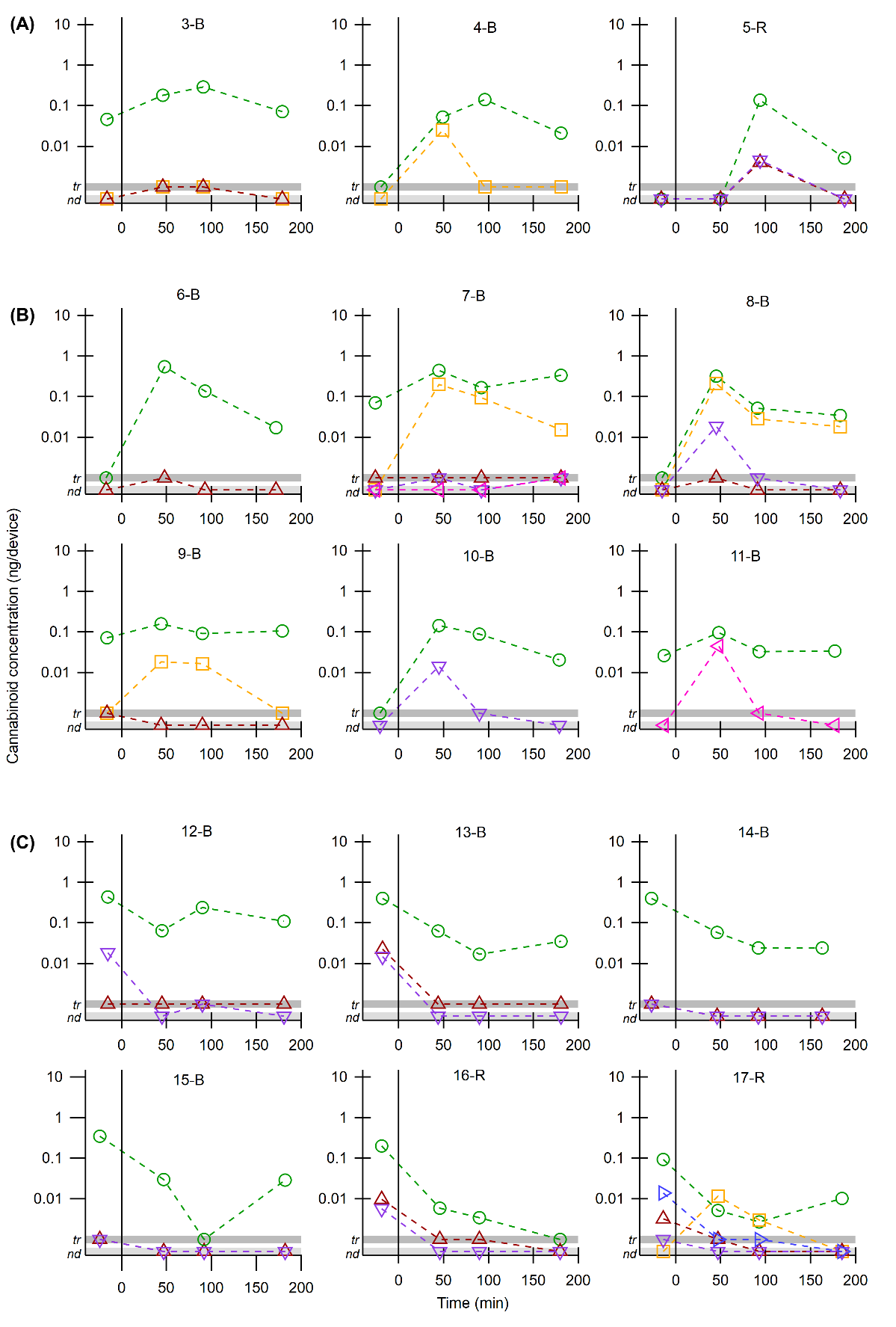


Figure S6. Cannabinoid concentration (ng/device) in breath before and after ingestion of a cannabis-infused edible on a log scale. Participants have different trends in THC concentration with **(A)** a maximum at 92 min after use, **(B)** a maximum at 47 min after use, and **(C)** a maximum THC concentration before ingestion. These participants are the same as shown in Figure 2 in the main text. THC are green circles, CBD are yellow squares, CBN are maroon upward-pointing triangles, CBG are downward-pointing purple triangles, CBC are pink leftward-pointing triangles, and THCA are rightward-pointing blue triangles.


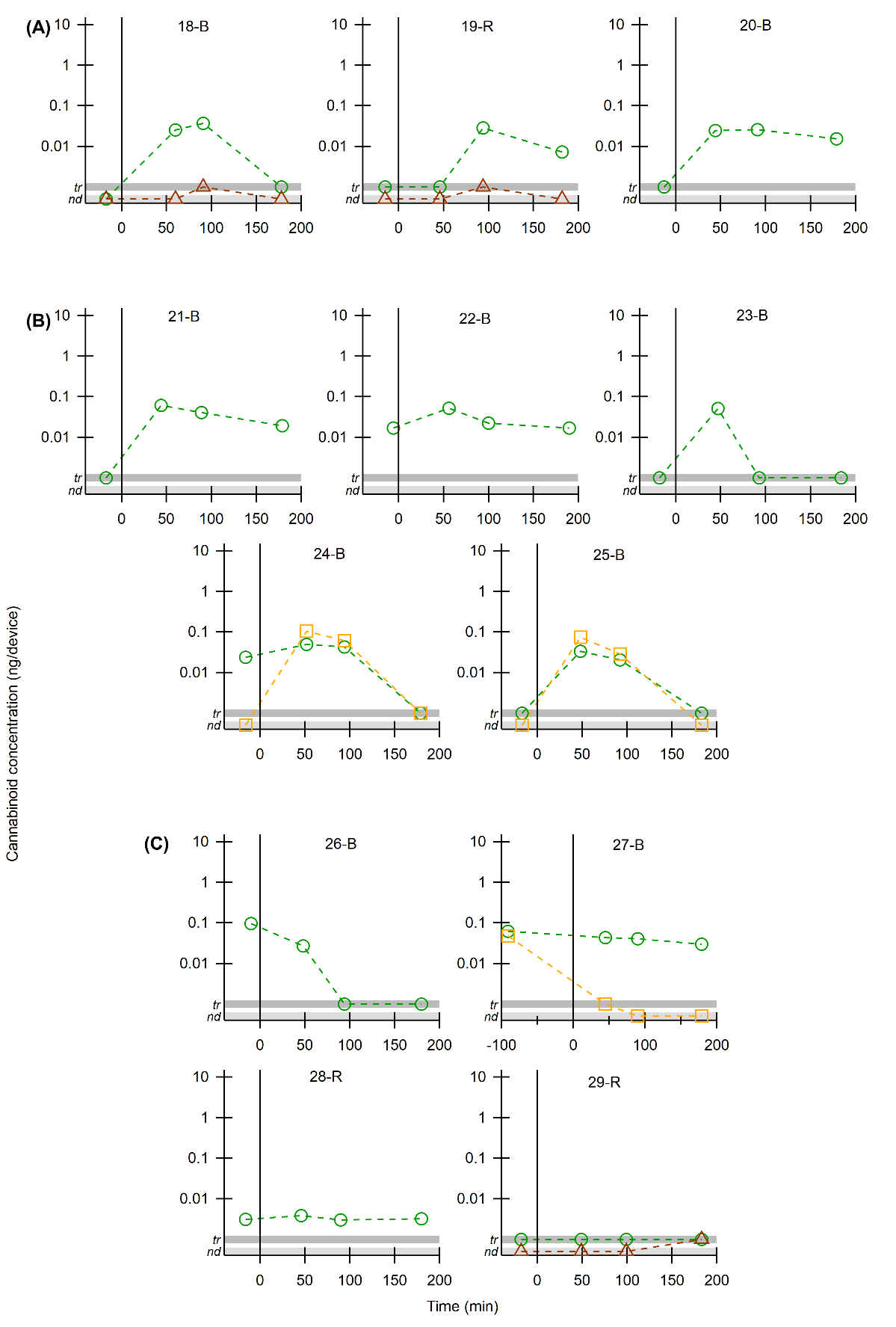


Figure S7. Cannabinoid concentration (ng/device) in breath before and after ingestion of a cannabis-infused edible on a log scale. Participants have different trends in THC concentration with **(A)** a maximum at 92 min after use, **(B)** a maximum at 47 min after use, and **(C)** a maximum THC concentration before ingestion. These participants are the same as shown in Figure 3 in the main text. THC are green circles, CBD are yellow squares, and CBN are maroon upward-pointing triangles.

**References**

1. Berry, J.L., et al., *Cannabinoids detected in exhaled breath condensate after cannabis use.* J Breath Res, 2024. **18**(4).

2. Henion, J., et al., *An analytical approach for on-site analysis of breath samples for Delta9-tetrahydrocannabinol (THC).* J Mass Spectrom, 2024. **59**(1): p. e4987.
